# Prefrontal Cortex Activation During Motor Sequence Learning Under Interleaved and Repetitive Practice: A Two-Channel Near-Infrared Spectroscopy Study

**DOI:** 10.3389/fnhum.2021.644968

**Published:** 2021-05-14

**Authors:** Maarten A. Immink, Monique Pointon, David L. Wright, Frank E. Marino

**Affiliations:** ^1^Sport, Health, Activity, Performance and Exercise (SHAPE) Research Centre, Flinders University, Adelaide, SA, Australia; ^2^Alliance for Research in Exercise, Nutrition and Activity, University of South Australia, Adelaide, SA, Australia; ^3^School of Exercise Science, Sport & Health, Charles Sturt University, Bathurst, NSW, Australia; ^4^Department of Health & Kinesiology, Texas A&M University, College Station, TX, United States

**Keywords:** motor sequence learning, contextual interference effect, interleaved practice, functional near infrared spectroscopy, prefrontal cortex (PFC), cognitive control

## Abstract

Training under high interference conditions through interleaved practice (IP) results in performance suppression during training but enhances long-term performance relative to repetitive practice (RP) involving low interference. Previous neuroimaging work addressing this contextual interference effect of motor learning has relied heavily on the blood-oxygen-level-dependent (BOLD) response using functional magnetic resonance imaging (fMRI) methodology resulting in mixed reports of prefrontal cortex (PFC) recruitment under IP and RP conditions. We sought to clarify these equivocal findings by imaging bilateral PFC recruitment using functional near-infrared spectroscopy (fNIRS) while discrete key pressing sequences were trained under IP and RP schedules and subsequently tested following a 24-h delay. An advantage of fNIRS over the fMRI BOLD response is that the former measures oxygenated and deoxygenated hemoglobin changes independently allowing for assessment of cortical hemodynamics even when there is neurovascular decoupling. Despite slower sequence performance durations under IP, bilateral PFC oxygenated and deoxygenated hemoglobin values did not differ between practice conditions. During test, however, slower performance from those previously trained under RP coincided with hemispheric asymmetry in PFC recruitment. Specifically, following RP, test deoxygenated hemoglobin values were significantly lower in the right PFC. The present findings contrast with previous behavioral demonstrations of increased cognitive demand under IP to illustrate a more complex involvement of the PFC in the contextual interference effect. IP and RP incur similar levels of bilateral PFC recruitment, but the processes underlying the recruitment are dissimilar. PFC recruitment during IP supports action reconstruction and memory elaboration while RP relies on PFC recruitment to maintain task variation information in working memory from trial to trial. While PFC recruitment under RP serves to enhance immediate performance, it does not support long-term performance.

## Introduction

Skill acquisition is enhanced by practice that exposes the learner to multiple task variations (Van Rossum, 1990; Shea et al., 2001). However, the extent to which variable practice benefits skill learning is dependent on how task variations are scheduled across practice (Shea and Morgan, 1979; Wright et al., 2016). According to Battig (1979), task variations can be scheduled such that the learner experiences either high or low levels of contextual interference. High contextual interference arises from interleaved practice (IP) where task variations are experienced in a random order across practice trials. In contrast, repetitive practice (RP), where task variations are practiced one at a time within a block of trials, establishes low contextual interference.

Battig (1979) proposed that relative to low contextual interference, high contextual interference would initially suppress practice performance but then enhance later performance. Termed the contextual interference effect, this phenomenon was first demonstrated in the motor learning domain by Shea and Morgan (1979). Here, the contextual interference effect was demonstrated as faster performance of movement sequences under RP than IP. In a delayed retention test, however, faster performance was observed in those who had experienced IP as opposed to RP. Since this seminal study, the contextual interference effect has been shown to be an enduring phenomenon that has been replicated with a variety of motor tasks and populations in both lab and applied settings (Magill and Hall, 1990; Brady, 1998; Pauwels et al., 2014; Wright et al., 2016; Immink et al., 2020).

### Theoretical Accounts of the Contextual Interference Effect

Delineation of the theoretical basis for the contextual interference effect has been elusive despite a high level of research attention devoted to this end (Wright et al., 2016). For some time, the contextual interference effect has been described as involving two general processes; elaboration (Shea et al., 1985; Shea and Zimny, 1988) and forgetting-reconstruction (Lee and Magill, 1983, 1985). According to the elaboration perspective (Shea et al., 1985; Shea and Zimny, 1988), IP allows for more elaborative processing of task information and thus the development of richer task representation. More elaborative processing is possible under IP because the exposure to multiple task variations within a succession of trials means that information from all the task variations can reside in working memory at any one time. This allows the learner to not only process features specific to the presently performed task variation but to also compare the present variation features with the features of other recently experienced task variations. Under RP, the learner is limited to processing information for one variation in isolation since working memory only holds information for one variation at a time.

Forgetting-reconstruction (Lee and Magill, 1983, 1985) describes differences in response planning that occur between IP and RP. Under IP, the learner is presented with a task variation that is distinct from that performed in the previous trial. As such, the response plan utilized in the previous trial, which would still reside in working memory, is not applicable to the present trial. The learner must thus forget the plan currently in working memory and reconstruct a new response plan according to the present task variation. This ongoing process of forgetting and reconstructing the action plan facilitates greater development of response planning processes, which affords long–term performance. Repetition of the same task variation under RP does little to encourage response planning development since the same response plan solution in working memory can be re-deployed across the trials involving the same task variation.

Initially, the elaboration and forgetting-reconstruction perspectives were viewed as opposing explanations to the contextual interference effect. However, as there has been evidence to support both perspectives (Brady, 1998; Immink and Wright, 2001), it appears more likely that elaboration and forgetting-reconstruction both contribute to the contextual interference effect (Immink and Wright, 2001). More generally, this suggests, as Battig (1979) originally proposed, that the contextual interference effect arises from a complex interaction of multiple performances and learning–related processes beyond those presently described by the elaboration and forgetting-reconstruction perspectives.

Despite the lack of a specific theoretical account for the contextual interference effect, there is common agreement that IP is more effortful and demanding than RP (Li and Wright, 2000; Wright et al., 2016; Immink et al., 2020). For example, Husak et al. (1991) reported higher physiological arousal under IP as compared to RP during the acquisition of a limb positioning task. Li and Wright (2000) demonstrated that IP places greater demands on attentional resources than RP. IP is thought to be more demanding because of how the high contextual interference practice format requires the learner to engage more extensively in the cognitive processes needed to acquire and perform novel action. This appears to be more so with respect to the processes associated with response preparation as opposed to processes associated with response execution.

Immink and Wright (1998) allowed learners to self-select the amount of time needed to plan a sequence of key presses prior to having to initiate and complete the sequence. Learners elected to use longer response preparation durations under IP conditions than under RP conditions, illustrating increased response preparation demands that occur under IP formats. In contrast to typical practice performance differences associated with the contextual interference effect, Immink and Wright (1998) reported comparable reaction time and movement time durations between the two practice conditions. This suggests that when the learner has the opportunity to complete the additional response preparation processes required under IP, they then exhibit no additional costs with respect to response execution processes relative to the learner experiencing RP (see also Immink and Wright, 2001).

### Neural Correlates of the Contextual Interference Effect

Findings from neuroimaging investigations into the contextual interference effect (Wright et al., 2016; Immink et al., 2020) lend further support to the notion that IP engenders more extensive involvement of response preparation processes than RP. The functional magnetic resonance imaging (fMRI) derived blood-oxygen-level-dependent (BOLD) signal has been shown to be higher during IP in several premotor cortical regions associated with response preparation, the dorsal and ventral premotor cortex (dPMC, vPMC, respectively), and the pre and proper supplementary motor area (preSMA, SMA, respectively; Cross et al., 2007; Wymbs and Grafton, 2009; Lin et al., 2011). The dPMC and vPMC are thought to be involved in the formation of stimulus-response associations while preSMA and SMA activity has been associated with the selection, retrieval, and organization of movement sequences (Gerloff et al., 1997; Verwey et al., 2002; Nachev et al., 2008). Increased premotor region activation and therefore, increased motor planning demands under IP provides a long-term benefit. Retention test performance is superior in those who previously experienced IP.

Importantly, the long-term performance benefit of IP is associated with reduced demands on response preparation processes. Specifically, Immink and Wright (1998) demonstrated that in the retention test, self-selected response planning time was shorter following IP than following RP. That IP affords lower response planning demands in the long–term is further supported by neuroimaging results. IP results in reduced neural recruitment in the premotor region during retention test performance when compared to those who previously practiced under a repetitive format (Lin et al., 2011; Wright et al., 2016; Immink et al., 2020).

Beyond the dPMC, vPMC, preSMA, and SMA, IP has been associated with increased neural recruitment in other motor planning regions including the inferior temporal lobe, angular gyrus, superior parietal lobe, precuneus, and the postcentral gyrus (Floyer-Lea and Matthews, 2005; Dayan and Cohen, 2011; Penhune and Steele, 2012; Hardwick et al., 2013; Immink et al., 2020). However, given that the contextual interference effect has been demonstrated in both verbal and motor learning domains (Magill and Hall, 1990), it would be expected that the neural correlates of the effect extend those specific to motor planning. Accordingly, regions associated with higher order, executive cognitive processes have also been demonstrated to be influenced by varying levels of contextual interference during practice. Specifically, Lin et al. (2011) reported increased bilateral prefrontal cortex (PFC) activity associated with IP. That increased prefrontal activity was not limited to the contralateral prefrontal region, relative to the response hand, suggests that the increased activity was not limited to motor learning processes but rather that high contextual interference required more extensive engagement in higher order cognitive processes.

The importance of executive control processes in the contextual interference effect is further highlighted by reports that across multiple practice sessions, IP exhibits increased activity in networks involving the dorsolateral prefrontal cortex (DLPFC) while IP and RP activation differences in networks involving premotor regions diminish (Lin et al., 2013). Moreover, DLPFC activation, but not premotor or motor region activation (Tanaka et al., 2010), during IP has been associated with performance benefits in retention and transfer test (Kantak et al., 2010, 2011; Lage et al., 2015). While Lin et al. (2011) reported increased bilateral prefrontal cortex in association with IP, others (Kantak et al., 2010, 2011; Tanaka et al., 2010; Lin et al., 2013) have only reported on differences with respect to the contralateral prefrontal region (Lage et al., 2015; Wright et al., 2016). As typically the non-dominant, left hand is used to investigate sequential motor learning it is difficult to ascertain if increased right DLPFC activity is specific to the effector limb employed in learning or if it is more generally related to the right hemisphere cortico-striatal network that has been described as the motor learning network (Rauch et al., 1995; Doyon et al., 1996).

To add further ambiguity, Lin et al. (2012) who investigated functional activation under high and low levels of contextual interference in young and older adults, reported increased recruitment associated with IP in the ipsilateral DLPFC. Moreover, RP exhibited higher activation in bilateral prefrontal regions than IP in older adults. This latter finding has similarly been reported by Pauwels et al. (2018) with a bimanual visuomotor task. Thus, currently, there are mixed reports of prefrontal region recruitment under IP and RP formats, which presents some limitations with respect to understanding the extent to which higher order cognitive processes contribute to the contextual interference effect.

### The Prefrontal Cortex as a Region of Interest for the Contextual Interference Effect

The prefrontal cortex is a region of particular interest to delineating a coherent theoretical account for the contextual interference effect given the central role of the top-down processes associated with this region in learning, memory, and performance (Shallice and Burgess, 1996; Smith and Jonides, 1999; Miller and Cohen, 2001). This is especially so as the complex regulation of cognitive control processes (Hommel, 2015) needed for optimal learning and performance is thought to be controlled by the more anterior regions of the prefrontal cortex (Jueptner et al., 1997; Koechlin et al., 2003; Koechlin and Summerfield, 2007; Badre et al., 2009). For example, regions within the prefrontal cortex are interconnected with the cingulate cortex, and the posterior parietal cortex as part of the default mode network is thought to facilitate the performance of novel or complex motor tasks (Petersen et al., 1998; Kelly and Garavan, 2005). Furthermore, the prefrontal cortex has also been associated with a number of processes that appear relevant to the effects of contextual interference on motor learning including performance strategy selection (Block et al., 2007), response inhibition (Verbruggen and Logan, 2008), interference control (Derrfuss et al., 2004) and working memory function (Cavanagh et al., 2018). With respect to the Li and Wright (2000) report of increased attention demands under IP, the prefrontal cortex is also associated with attention regulation (Banich et al., 2000; Milham et al., 2003). Not only is the prefrontal cortex relevant to practice related effects of IP and RP, but its function also appears to be relevant to the effects observed in retention as the prefrontal cortex contributes to memory retrieval (Tomita et al., 1999) and memory consolidation processes (Muellbacher et al., 2002).

### Functional Near Infrared Spectroscopy of the Prefrontal Cortex Under High and Low Contextual Interference

While previous neuroimaging studies addressing the contextual interference effect for motor skill learning have mostly relied on fMRI, the present work implemented functional near infrared spectroscopy (fNIRS) as the latter neuroimaging methodology offers higher temporal resolution and allows neural recruitment to be assessed based on independent changes in oxygenated (HbO_2_) and deoxygenated (Hb) hemoglobin (Cui et al., 2011; Ayaz et al., 2019). In addition, fNIRS allows continuous measurement of HbO_2_ and Hb during task performance (Obrig et al., 1996; Villringer and Chance, 1997; Cui et al., 2010, 2011). fNIRS has been previously employed to assess prefrontal recruitment associated with motor learning (e.g., Hatakenaka et al., 2007; Leff et al., 2008; Ayaz et al., 2011; Goto et al., 2011) and more generally, prefrontal cortex recruitment associated with cognitive task difficulty and mental workload (Ayaz et al., 2012; Sato et al., 2013). Specific to the contextual interference effect, Shewokis et al. (2017) previously assessed bilateral PFC recruitment, including DLPFC and medial PFC regions, with fNIRS during surgical skills training under RP and IP conditions. Based on changes in total hemoglobin, a composite of oxygenated and deoxygenated hemoglobin changes, a mixed pattern of practice schedule-dependent differences in PFC recruitment at training were reported. Consistent with previous fMRI work demonstrating increased PFC recruitment under IP (e.g., Lin et al., 2011), mean change in total hemoglobin was higher under IP with surgical camera navigation and dissection tasks. However, with lifting and grasping surgical task, the mean change in total hemoglobin was higher under RP; this latter finding being consistent with (Pauwels et al., 2018). Shewokis et al. (2017) also evaluated PFC recruitment during test performance of the trained surgical tasks. Here, hemisphere-specific differences between RP and IP were reported for mean changes in total hemoglobin. While the practice groups did not differ with respect to total hemoglobin changes, those trained under RP exhibited significantly higher mean changes in total hemoglobin in the right PFC. That RP resulted in increased recruitment of only the right PFC at test differs from the bilateral increases in PFC recruitment following RP reported in fMRI work (see Immink et al., 2020).

A previous comparison of fNIRS and fMRI measures of prefrontal activity with a working memory task revealed a positive correlation between oxygenated hemoglobin concentration and the BOLD signal and a negative correlation between deoxygenated hemoglobin concentration and the BOLD signal (Huppert et al., 2006; Cui et al., 2011; Sato et al., 2013; Maggioni et al., 2015; Wijeakumar et al., 2017). Changes in oxygenated and deoxygenated hemoglobin concentrations occur as a result of neural activity through a process as neurovascular coupling (Causse et al., 2017). Neurovascular coupling results in an oversupply of oxygenated blood which causes oxygenated hemoglobin to increase and deoxygenated hemoglobin to decrease (Causse et al., 2017; Ayaz et al., 2019). While neuronal activity is typically associated with coupled changes in oxygenated and deoxygenated hemoglobin, it has been shown that under increased neural demands, changes in oxygenated and deoxygenated hemoglobin concentrations can be temporarily decoupled (Tam and Zouridakis, 2014, 2015). For example, increased metabolic demands associated with elevated neural activity can result in no change or a decrease in oxygenated hemoglobin along with an increase in deoxygenated hemoglobin concentration (Tam and Zouridakis, 2015). For the purpose of the present study, increased prefrontal cortex region oxygenated hemoglobin or decreased deoxygenated hemoglobin concentrations, relative to rest, were interpreted as representing increased neural recruitment of the prefrontal cortex.

### The Present Work

The forgetting-reconstruction (Lee and Magill, 1983, 1985) and elaboration (Shea et al., 1985; Shea and Zimny, 1988) perspectives of the contextual interference effect along the evidence of increased attention demands (Li and Wright, 2000) and more extensive motor planning processes (Immink and Wright, 1998, 2001) under IP would collectively lend to the prediction that IP would exhibit increased levels of prefrontal cortex recruitment relative to RP based on the concept of heightened cognitive demand under high contextual interference (Wright et al., 2016). However, an alternative prediction is that RP requires greater or equivalent recruitment of the prefrontal cortex (Lin et al., 2012; Pauwels et al., 2018). RP may place increased demands on attentional and working memory processes due to the need to maintain target information from trial to trial (Lin et al., 2012; Wright et al., 2016). Whether the increased prefrontal activity is bilateral or is specific to the right or left hemisphere is difficult to predict at this stage given heterogeneity in reports from previous neuroimaging work addressing the contextual interference effect. Here, we aim to address the limitations associated with unimanual tasks by the inclusion of a key-pressing task involving both left and right hands.

## Materials and Methods

### Participants

Twenty-six adults (age 22.9 ± 5.2 years, 14 females) participated in the present study. The sample size estimate was based on an effect size of Cohen’s *f* = 0.29 (*d* = 0.57) reported for the CI effect in basic research (Brady, 2004). With G*power (version 3.1.9.7; Faul et al., 2007), we estimated a total sample size of 26 for a repeated-measures analysis of variance (ANOVA) design with within-between interactions, two groups, and two measures (response time at training and test) based on *p* < 0.05 and 0.80 power. This sample size resulted in group sizes which were larger (*N* = 13 vs. *N* = 5) to a previous fNIRS investigation of fine motor skills under contextual interference conditions (Shewokis et al., 2017). All participants were classified as right-hand dominant based on the Edinburgh Handedness Inventory—Short Form (Oldfield, 1971; Veale, 2014) and all reported no history of neurological, visual, neuromuscular, cognitive, mental health, or musculoskeletal conditions. Participants were naïve to the purpose of the study. The study was approved by Charles Sturt University Human Research Ethics Committee and all participants provided written informed consent.

### Sequence Learning Task and Apparatus

The sequence learning task consisted of five-key press sequences based on six response keys that were located on a standard QWERTY keyboard that was modified such that all keys were removed, except for the S, D, F, J, K, and L keys (Immink and Wright, 1998). For the purpose of the task, these keys were numbered from left to right as 1, 2, 3, 4, 5, and 6. The ring, middle, and index fingers of the left hand depressed the 1, 2, and 3 keys, respectively, while the index, middle and ring fingers of the right hand depressed the 4, 5, and 6 keys, respectively. The sequence learning task was programmed on the E-Prime 2.0 Professional Edition (Psychology Software Tools, Sharpsburg, PA) platform. Task stimuli were presented on a 48 cm CRT monitor with an 85 Hz refresh rate. Participants were positioned about 60 cm from the monitor, though this distance was not strictly enforced. Each character in the sequence presentation transcended 1.91 degrees of visual angle.

**Figure 1:**
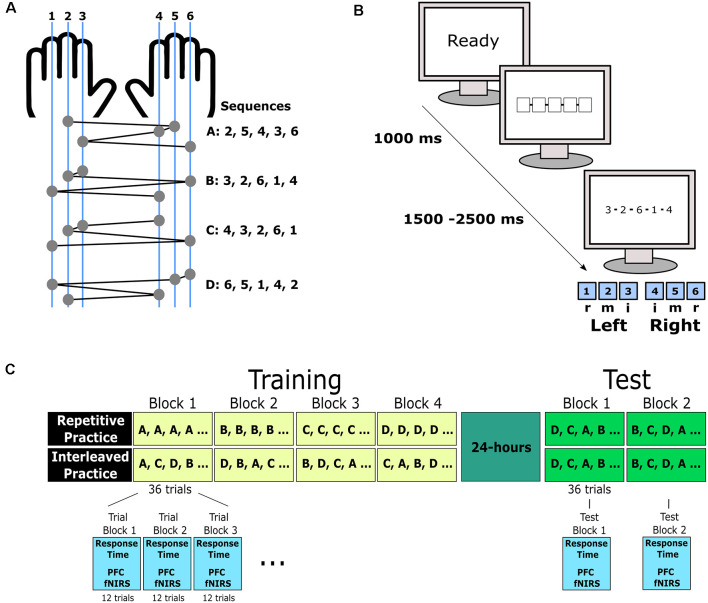
Sequence learning involved four bimanual five-item key press sequences **(A)**. In each training and test trial, a “Ready” warning signal preceded a fixation stimulus involving five boxes. After a random interval, the response stimulus was presented based on a sequence of numbers that represented the order that each of the keys was to be pressed. The five keys were numbered one to five from left to right on the keyboard. Keys were pressed by either the index (i), middle (m), or ring (r) finger of the left and right hand **(B)**. The four sequence variations were trained across four training blocks. Under repetitive practice, one sequence variation was practiced in each training block, whereas for interleaved practice, all four sequences were performed in a block following a pseudo-random order given a sequence could not be repeated in consecutive trials. The order of sequences under RP was counterbalanced. The training involved four blocks of 36 trials. For the purpose of analysis, each block was divided into three trial blocks consisting of 12 trials each. This resulted in 12 trial blocks for analysis. Approximately 24 h after training completion, participants completed two test blocks of 36 trials each. For the test, the four sequence variations were presented in a pseudo-random order **(C)**.

Each trial of the sequence learning task commenced with the presentation of a “READY” message in the middle of the viewing screen for 1,000 ms (see Figure 1B). This was then replaced with a fixation stimulus comprised of five unfilled boxes placed in the center of the screen to indicate the location where sequence information would be presented. The fixation stimulus remained on the screen for a 1,500–2,500 ms random foreperiod. Next, the fixation stimulus was replaced by a sequence of five numbers (e.g., 3, 2, 6, 1, 4) that indicated the order of keypresses (e.g., left index, left middle, right ring, left ring, right index). The response stimulus remained on the screen until the sequence was completed. After each key press, a solid white box appeared under the corresponding position within the sequence. Immediately after the fifth key press, the participant was presented with trial feedback for 2,000 ms that indicated whether the entered sequence was correct (five keys pressed in the target order) or incorrect. For correct responses, the participant was provided with feedback about response time, calculated as the elapsed time between response stimulus presentation and the fifth keypress, in seconds. For each trial, response accuracy, where all five key presses matched the target sequence, and response time, the latency between response stimulus presentation and the fifth key press, was measured. Participants were instructed that response accuracy and response time were equally important for the task. The sequence learning task is illustrated in Figure 1.

### Near-Infrared Spectroscopy (NIRS)

A 2-channel, continuous-wave NIRS instrument (Oxymon MKII, Artinis Medical Systems B.V., Zetten, The Netherlands) was used to examine changes in oxygenated (HbO_2_) and deoxygenated (HbR) hemoglobin in the PFC throughout resting baseline, sequence learning and test. HbO_2_ and HbR concentrations (in micro Molar, μM, units) were calculated using a modified Beer-Lambert law using proprietary software and based on the absorption coefficient of continuous wavelength infra-red light (856 and 764 nm) and age-dependent differential path-length factors (range: 5.76–5.85; Duncan et al., 1996; Billaut et al., 2010). An emitter-optode pair (a light emitter and three separate photoreceptors) with an inter-optode distance of 35 mm (Derosière et al., 2014) was placed at the midpoint between Fp1 and F3 landmarks for the left PFC and a second emitter-optode pair was placed at the midpoint between Fp2 and F4 landmarks for the right prefrontal cortex based on the international EEG 10-20 system (Perrey, 2008). Previously, MRI scans have demonstrated that these sensor placement locations overlie the dorsolateral and orbitofrontal regions of the prefrontal cortex (Tanida et al., 2007). The 35 mm inter-optode distance was within the range recommended by Wang et al. (2019) for optimal measurement of brain hemodynamics. Placement sites were measured from the medial tip of the eyebrow and then cleaned with an alcohol swab. Optode placement sites were then marked with an indelible pen to standardize optode positioning between practice and test sessions. Optodes were affixed to the skin with double–sided self-adhesive disks and a black elastic headband was worn over the probes to further secure placement and minimize the effects of ambient light. NIRS data were recorded at 10 Hz. In conjunction with the NIRS instrument, Oxysoft software (Artinis Medical Systems B.V., Zetten, The Netherlands) allowed real-time visualization of signals from each channel. Prior to recording, the quality of the signal was inspected to ensure an acceptable signal to noise ratio was obtained based on the light source strength and receiver gain. Upon reaching a quality signal from each channel, a zero baseline was set and recording commenced.

Oxysoft was used to apply kernel smoothing with a Gaussian filter, inspect for the presence of movement artifacts and then export raw NIRS data from rest, training, and test phases. Raw data was then imported into a customized LabVIEW program (National Instruments, Austin, TX, USA) to extract data based on training and test epochs which corresponded to training and test trial blocks. Epochs were identified with synchronized markers, which were automatically triggered by E-Prime into Oxysoft. For training, markers were synchronized at the start of trials 1, 13, and 25, and the completion of trial 36 within each block. This resulted in three epochs per training block for a total of 12 training trials blocks across the 4 training blocks. For the test, markers were synchronized at the start of trial 1 and the end of trial 36 in each block, resulting in two epochs across the 2 test blocks. For each epoch as well as resting-state recordings, the LabVIEW program calculated average HbO_2_ and HBR concentrations.

### Procedure

Each participant attended the laboratory for two experimental sessions separated by approximately 24 h where the first session involved sequence learning and the second session involved a test of practiced sequences. After providing consent, participants were seated and were then provided with an overview of the procedure. Then, NIRS recording sites were measured, cleaned, and marked before the probes were fitted in place. Participants then remained still, did not talk and had their eyes closed for a 2 min period while resting baseline cerebral oxygenation was recorded. Participants were then randomly allocated to undertake sequence learning under either repetitive or IP schedules. All participants first received task familiarization based on written instructions and visual examples for how the stimuli related to responses. Participants had the opportunity to ask for verbal clarification of the task instructions if needed. Once participants were satisfied with the task instructions, they initiated a bout of sequence practice involving four trial blocks consisting of 36 trials each. The practice involved four sequences (A: 2, 5, 4, 3, 6; B: 3, 2, 6, 1, 4; C: 4, 3, 6, 2, 1; D: 6, 5, 1, 4, 2; see Figure 1A) that involved a different key press at the start of each sequence, three inter-hand changes and one change to a neighboring key. Under IP, all four sequences were practiced within each trial block following a pseudorandomized order for a cycle of four trials with the condition that each sequence was practiced once in each cycle and a sequence was not repeated between cycles. Under RP, only one sequence was practiced within each trial block. The order of sequence presentation was randomized between participants in the RP condition. A 120 s rest interval was provided to all participants between trial blocks (see Figure 1C). During each trial block, left and right PFC hemodynamics was continuously recorded using two-channel NIRS. Participants were instructed to not move their head or body during task performance.

Session two was designed as a delayed, retention test of performance based on prior training conditions. Participants completed two test blocks consisting of 36 trials with the previously practiced sequences using an interleaved schedule. Left and right PFC hemodynamic responses during each test block were continuously recorded using NIRS. Block and rest period durations matched training conditions, however, performance feedback was not provided after each trial.

### Statistical Analysis

Analysis of response error rates revealed no significant group differences at training, *F*_(1,24)_ = 0.001, *p* = 0.97, or test, *F*_(1,24)_ = 1.53, *p* = 0.23). Overall, error trials occurred in 10.1% of the training trials and 8.0% of the test trials. Given the absence of group differences in performance accuracy at training and test as well as the absence of significant correlation between participant error rate and response time at training (*p* = 0.33) and test (*p* = 0.53), analysis of sequence performance was based on response time.

To analyze training response time performance, the four blocks were each divided into three trial blocks reflecting performance on the first, middle and last 12 trials of each block. For example, trial block one included trials 1–12 of block one, trial block two included trials 13–24 of block one, trial block three included trials 25–36 of block one, and so on. This resulted in a total of 12 trial blocks for training. For each participant, the mean response time was calculated for all accurate trials in each of the 12 trial blocks. The training mean response time was submitted to a 2 (training schedule: repetitive, interleaved) × 12 (trial blocks 1–12) ANOVA with repeated measures on the latter factor. To analyze response time performance at test, the mean response time of accurate trials was calculated for each participant and each test block. Test mean response time was submitted to a 2 (training schedule: repetitive, interleaved) × 2 (test blocks one-two) ANOVA with repeated measures on the latter factor. We assessed changes in response time between the end of training and the first test block. For this, the percentage change in mean response time was first calculated for each participant based on the difference between test block 1 and training block 12 values as a ratio of training block 12 response time. Participant response time percentage change was then submitted to univariate ANOVA with the training group as the between-subject factor. Separate one sample *t*-tests were conducted for IP and RP groups to determine if the percentage change in response time significantly differed from zero.

Mean HbO_2_ and HbR NIRS values were separately calculated for each participant, each sensor, and each trial block. Participant mean values were then normalized against corresponding right and left HbO_2_ and HbR NIRS 120-s resting baseline values (Ayaz et al., 2019). This resulted in right and left <HbO_2_ and <HbR values reflecting changes in HbO_2_ (<[HbO_2_]) and HbR (<[HbR]) concentrations (μM) in each training trial block relative to resting baseline. The training left and right PFC <[HbO_2_] and <[HbR] values were separately submitted to 2 (training schedule: repetitive, interleaved) × 2 (sensor: right, left) × 12 (trial blocks 1–12) ANOVA with repeated measures on the last two factors. For the test, right and left <[HbO_2_] and <[HbR] were calculated based on participant mean right and left HbO_2_ and HbR NIRS values for test block one and 2 30-s epochs, which were then normalized against resting baseline HbO_2_ and Hb NIRS. Test <[HbO_2_] and <[HbR] values were separately submitted to 2 (training schedule: repetitive, interleaved) × 2 (sensor: right, left) × 2 (test blocks 1–2) ANOVA with repeated measures on the last two factors.

In all ANOVA, Greenhouse–Geisser degrees of freedom correction was applied when Mauchly’s test did not confirm the sphericity assumption. For clarity of communication, the original degrees of freedom are presented along with corrected *F* test and *p*-values. Significant main effects of the trial block at training or significant group × trial block interactions were evaluated using *post-hoc* pair-wise comparisons with least square difference adjustment. The effect size of any significant main effects or interactions was calculated as partial eta squared ($\eta_{p}^{2}$).

## Results

### Training

#### Sequence Performance

**Figure 2:**
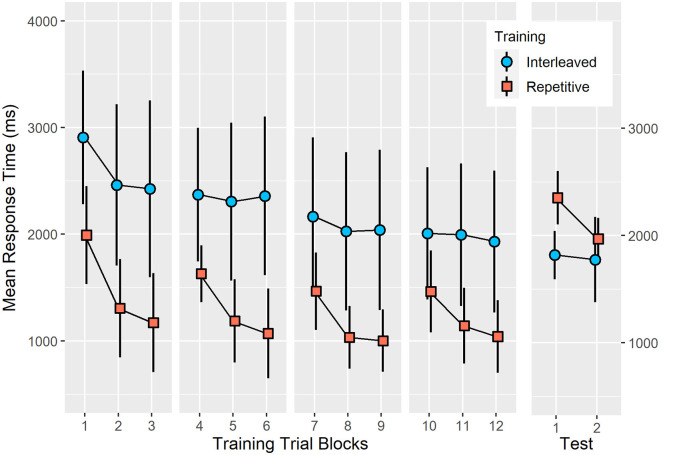
Mean response time of accurate bimanual five-key press sequences during training under interleaved or RP schedules and a 24-h delayed test. The pattern of results was consistent with the contextual interference effect (Shea and Morgan, 1979) since IP resulted in a significantly longer mean response time at training (*p* < 0.001, $\eta_{p}^{2}$ = 0.53) but resulted in a significantly shorter mean response time at test (*p* < 0.001, $\eta_{p}^{2}$ = 0.35). Error bars represent standard deviation.

Analysis of training mean response time (see Figure 2) revealed a significant group effect (*F*_(1,24)_ = 27.27, *p* < 0.001, $\eta_{p}^{2}$ = 0.53), training block effect (*F*_(11,264)_ = 36.80, *p* < 0.001, $\eta_{p}^{2}$ = 0.61) and a significant group × training block interaction (*F*_(11,264)_ = 6.78, *p* < 0.001, $\eta_{p}^{2}$ = 0.22). In all training trial blocks, IP demonstrated significantly longer response times than RP (all *p* < 0.05). The source of the interaction related to different profiles of trial block changes between the two training conditions. For example, under repetitive training, the mean response time was not significantly different between trial blocks 3, 6, 9, and 12 as well as between trial blocks 4, 7, and 10. In contrast, under IP, mean response time was not significantly different between trial blocks two to six and then between trial blocks eight to 12.

#### Hemoglobin Concentration Changes

**Figure 3:**
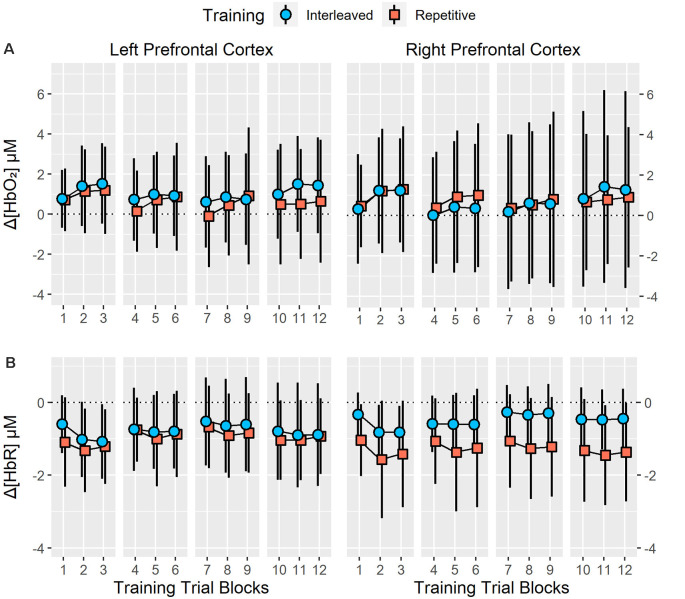
Resting state to task related changes in right and left prefrontal cortex (PFC) oxygenated (Δ[HbO_2_]; **A**) and deoxygenated ([ΔHbR]; **B**) hemoglobin concentration during 12 training trial blocks with four bimanual five-key press sequences under interleaved or repetitive training schedules. Analysis revealed no significant main effect or interactions of training groups on right and left PFC Δ[HbO_2_] or Δ[HbR] (all *p* > 0.20). Error bars represent standard deviation.

Analysis of training Δ[HbO_2_] revealed no significant main effect of group (*p* = 0.85), sensor (*p* = 0.87) or trial block (*p* = 0.13) as well as no significant group × sensor (*p* = 0.68), group × trial block (*p* = 0.64), sensor × trial block (*p* = 0.65) or group × sensor × trial block (*p* = 0.60) interactions. For training Δ[HbR], a significant main effect of trial block was identified, *F*_(11,264)_ = 4.35, *p* < 0.01, $\eta_{p}^{2}$ = 0.15. However, no significant main effect of group (*p* = 0.17) or sensor (*p* = 0.95) as well as no significant group × sensor (*p* = 0.23), group × trial block (*p* = 0.73), sensor × trial block (*p* = 0.52) or group × sensor × trial block (*p* = 0.27) interactions were revealed. *Post-hoc* analysis of the trial block main effect indicated that Δ[HbR] significantly decreased from trial block one to trial blocks two and three (*p* < 0.05) but no significant changes in Δ[HbR] were observed between trial blocks four to six, seven to nine or 10–12. At trial block 12, Δ[HbR] was significantly higher than in trial blocks two and three (*p* < 0.05) but was significantly lower than in trial blocks seven and nine (*p* < 0.05). Training Δ[HbO_2_] and Δ[HbR] are presented in Figure 3.

### Test

#### Sequence Performance

Analysis of mean response time at test (see Figure 2) revealed a significant group effect (*F*_(1,24)_ = 13.00, *p* < 0.001, $\eta_{p}^{2}$ = 0.35), test block effect (*F*_(1,24)_ = 27.27, *p* < 0.0001, $\eta_{p}^{2}$ = 0.53) and a significant group × test block interaction (*F*_(1,24)_ = 17.37, *p* < 0.0001, $\eta_{p}^{2}$ = 0.42). In the first test block, interleaved training (*M* = 1,818.3 ms, SEM = 65.8) resulted in significantly shorter mean response time than following repetitive training (*M* = 2,352.2 ms, SEM = 65.8; *p* < 0.0001). In test block two, however, mean response time did not significantly differ between interleaved (*M* = 1,775.3 ms, SEM = 86.7) and repetitive training groups (*M* = 968.9 ms, SEM = 86.7; p = 0.13).

Response time percentage change between training block 12 and test block one was significantly different between interleaved (*M* = − 7.00%, SEM = 9.52) and repetitive (*M* = 55.72%, SEM = 3.93) training groups, *F*_(1,24)_ = 37.05, *p* < 0.0001, $\eta_{p}^{2}$ = 0.61). A significant percentage increase in response time occurred following repetitive training (*p* < 0.001) while there was no significant change in response time following interleaved training (*p* = 0.48).

#### Hemoglobin Concentration Changes

**Figure 4:**
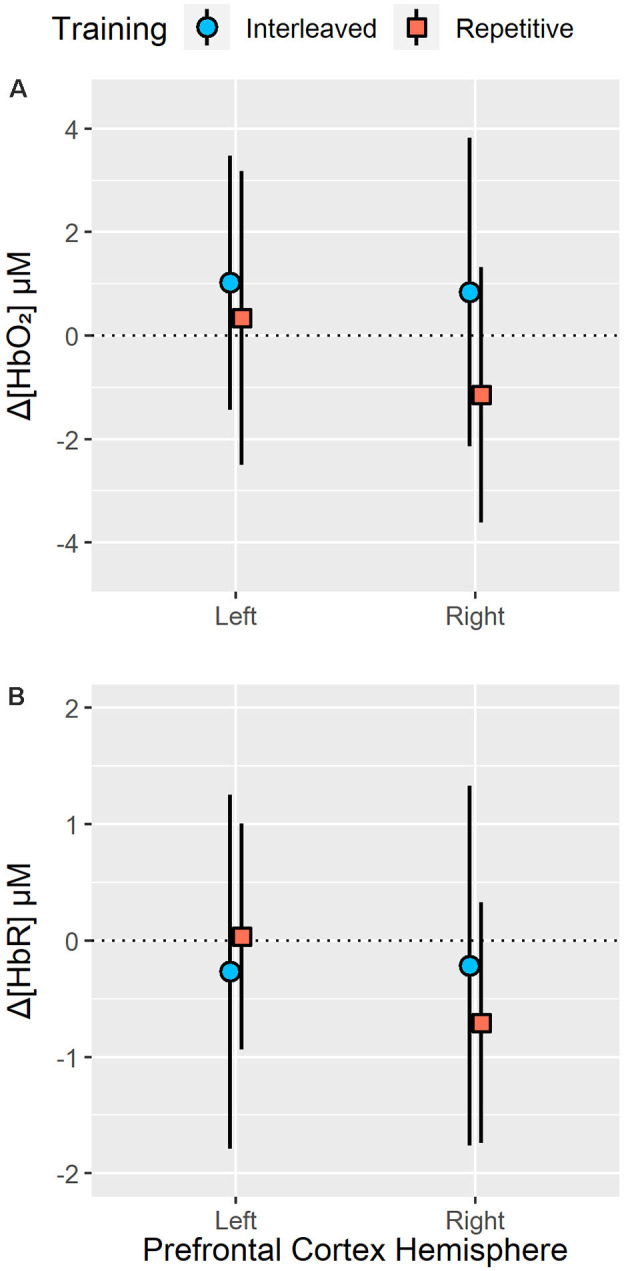
Resting state to task related changes in right and left PFC oxygenated (Δ[HbO_2_]; **A**) and deoxygenated (Δ[HbR]; **B**) hemoglobin concentration during a 24-h delayed test of bimanual five-key press sequences acquired under interleaved or repetitive training schedules. Analysis of right and left PFC Δ[HbO_2_] revealed no significant main effect or interactions of training groups (all *p* > 0.10). In contrast, analysis revealed a significant group × sensor (left or right PFC hemisphere) interaction (*p* < 0.05, $\eta_{p}^{2}$ = 0.15) for Δ[HbR] at test. While interleaved training did not result in any hemispheric differences in Δ[HbR], repetitive training resulted in significantly lower Δ[HbR] in the right PFC (*p* < 0.05). Error bars represent standard deviation.

No significant main effect of group (*p* = 0.17), sensor (*p* = 0.08) or test block (*p* = 0.34) as well as no significant group × sensor (*p* = 0.16), group × test block (*p* = 0.45), sensor × test block (*p* = 0.93) or group × sensor × test block (*p* = 0.87) interactions were observed for Δ[HbO_2_]. Analysis of test Δ[HbR] revealed a significant group × sensor interaction, *F*_(1,24)_ = 4.31, *p* < 0.05, $\eta_{p}^{2}$ = 0.15 (see Figure 4). Pair-wise analysis indicated no significant differences (*p* > 0.35) in Δ[HbR] between interleaved and repetitive training group in the left (interleaved training *M* = −0.27, SEM = 0.35; repetitive training *M* = 0.035, SEM = 0.35) or right hemispheres (interleaved training *M* = −0.22, SEM = 0.37; repetitive training *M* = −0.71, SEM = 0.37). However, for the repetitive training group, Δ[HbR] was significantly lower in right hemisphere than the left hemisphere (*p* < 0.05) while Δ[HbR] did not significantly differ between hemispheres in the interleaved training group (*p* = 0.86). No significant main effects of group (*p* = 0.84), sensor (*p* = 0.08), test block (*p* = 0.89), group × test block (*p* = 0.42), sensory × test block (*p* = 0.50) or group × sensor × test block (*p* = 0.30) interactions were observed for Δ[HbR].

## Discussion

A unifying theme underlying current explanations for the contextual interference effect is that IP requires the learner to engage more extensively in cognitive processes associated with motor skill learning and performance (Wright et al., 2016). An extension of this theme is that IP exposes the learner to increased demands that are observed behaviorally as suppressed practice performance (Shea and Morgan, 1979), physiologically as elevated arousal (Husak et al., 1991), and neurophysiologically as increased neural recruitment of motor planning regions (Lage et al., 2015; Wright et al., 2016; Immink et al., 2020). The Li and Wright’s (2000) demonstration of increased attention demands under IP would also suggest that high contextual interference requires further engagement of higher order cognitive processes that beyond motor skills are more generally associated with goal-oriented behavior.

Neuroimaging findings support this view as increased recruitment of the prefrontal cortex has been associated with IP (Kantak et al., 2010, 2011; Tanaka et al., 2010; Lin et al., 2011, 2013). However, neuroimaging work addressing the contextual interference effect presents some contradictory evidence with respect to recruitment in the prefrontal region. This includes reports of increased prefrontal recruitment under RP (Lin et al., 2012; Pauwels et al., 2018). Due to this discrepancy, the aim of the present study was to revisit prefrontal region activation during and following practice with high and low contextual interference using fNIRS neuroimaging.

### Performance and Prefrontal Cortex Recruitment During Practice Under High and Low Contextual Interference

As expected from the contextual interference effect, the IP format exhibited longer sequence completion durations than RP during training. Prefrontal region recruitment associated with these performance differences was evaluated with two-channel fNIRS recording of the left and right prefrontal region. For this, increased neural recruitment was interpreted from either increases in oxygenated hemoglobin concentration or decreases in deoxygenated hemoglobin concentration (Huppert et al., 2006; Cui et al., 2011; Sato et al., 2013; Maggioni et al., 2015; Wijeakumar et al., 2017). Relative to resting conditions, both practice formats exhibited bilateral increases in oxygenated hemoglobin concentration and decreased deoxygenated hemoglobin concentration. This is reflective of the expected increase in prefrontal region recruitment associated with acquiring and executing the sequencing task. Critically though, there were no observed significant group main effects or interactions for both oxygenated and deoxygenated hemoglobin concentration changes. Thus, the present results suggest that high and low contextual interference practice conditions required equivalent levels of prefrontal region recruitment.

Equivalent prefrontal recruitment under IP and RP is at odds with previous fMRI-based reports of increased prefrontal recruitment of the right hemisphere (Kantak et al., 2010, 2011; Tanaka et al., 2010; Lin et al., 2011, 2013), under either IP (Lin et al., 2012; Pauwels et al., 2018) or RP (Lin et al., 2012; Pauwels et al., 2018). At this stage, direct comparison of the present findings with previous reports might be somewhat premature given that the present work employed the NIRS technique whereas previous work used the fMRI BOLD signal to infer neural recruitment. It is thus not possible to rule out that divergence in these findings is due to differences in how neural recruitment is inferred under each neuroimaging technique. Nevertheless, the present training findings were also not consistent with Shewokis et al. (2017) who employed fNIRS methodology to investigate PFC recruitment under high and low contextual interference with surgical motor skills. There, higher PFC total hemoglobin was associated with IP during the performance of surgical camera navigation and dissection tasks whereas RP elicited higher PFC total hemoglobin with a lifting and grasping surgical task. Thus, a comparison of the present PFC recruitment findings with previous reports from both fMRI BOLD and fNIRS total hemoglobin highlights a high degree of uncertainty with respect to how high and low contextual interference influences prefrontal region recruitment. Further investigation is necessary to resolve this montage of reports of PFC recruitment during IP and RP. One consideration for future work is the possibility that PFC recruitment under practice conditions with high and low contextual interference might be task-specific. For example, as PFC recruitment has been shown to depend on sensory input, action rules and abstraction, and task performance feedback, and reward (Rao et al., 1997; Duncan, 2001; Freedman et al., 2001; Cools et al., 2004), task differences in any of these characteristics might result in differential patterns of PFC recruitment between neuroimaging studies.

As they are, the present findings suggest that IP and RP formats impart equivalent levels of prefrontal region recruitment, at least with respect to the acquisition of discrete key-press sequences. However, retention test performance and prefrontal hemodynamics suggest that while IP and RP might involve comparable levels of prefrontal recruitment, the processes underlying this recruitment might be quite distinct under the two practice formats.

### Long-Term Performance and Prefrontal Cortex Recruitment Following High and Low Contextual Interference Practice Conditions

The significant group effect for response duration in the retention test was consistent with what would be expected from the contextual interference effect. Exposure to high contextual interference under IP provided for enhanced retention performance when compared to the long-term outcomes from RP. Enhanced retention performance exhibited by IP was based on stabilization of the motor sequence representation in the 24-h period between training and test. This was illustrated by equivalent response time values between the final training block and the first test block. Following RP, in contrast, there was a significant increase in response time over the 24-h period, suggesting that low contextual interference provides for less stabile representation of the practiced motor sequence. This finding is consistent with previous demonstrations that IP establishes stability in motor memory representations while RP typically results in forgetting in the period following training (e.g., Kim et al., 2018).

Evaluation of prefrontal area hemodynamics associated with retention performance revealed no significant differences in oxygenated hemoglobin concentration changes between the two practice groups. With respect to deoxygenated hemoglobin concentration dynamics, however, a significant group by sensor interaction was observed during retention performance. This interaction was based on hemispheric differences in deoxygenated hemoglobin concentration changes following RP but not following IP. Specifically, those who had practiced under the RP exhibited a greater reduction of deoxygenated hemoglobin concentration in the right prefrontal area than the left. These hemispheric differences in deoxygenated hemoglobin dynamics illustrate that along with deficits in long-term performance, those who had practiced under low contextual interference relied on greater recruitment of the right prefrontal hemisphere during the retention test. The IP group, on the other hand, exhibited equivalent changes in deoxygenated hemoglobin concentration between the left and right prefrontal areas. Thus, concomitant with enhanced retention performance, those who had practiced under high contextual interference exhibited uniform recruitment of the left and right prefrontal regions.

That RP results in elevated recruitment of the right prefrontal cortex during retention test performance is consistent with the previous findings from work employing fMRI (Lin et al., 2011) and fNIRS (Shewokis et al., 2017) approaches to quantifying PFC recruitment following IP and RP. It should be noted that (Pauwels et al., 2018) did not report any contextual interference differences in prefrontal region recruitment during delayed retention although their study involved a bimanual visuomotor task, which is different from the key-press sequencing task employed in the present work. As with discrepancies in PFC recruitment under IP and RP formats, inconsistencies in PFC recruitment at test might be attributed to task specificity. Moreover, mixed test findings might be associated with differences in participant age given that reports have been based on young and old adult population (Lin et al., 2012; Pauwels et al., 2018). Future work is needed to clarify what appears to be a discrepancy in current reports of prefrontal cortex recruitment during retention test performance following high and low contextual interference practice conditions. As part of this, some attention should be devoted to delineating the extent to which prefrontal cortex recruitment following IP and RP is influenced by the age of the learner. For now, as the present findings are comparable to those reported in Lin et al. (2011), it seems is appropriate to undertake some consideration of what might underlie increased reliance on the right prefrontal cortex following RP but not IP. However, such explanation might not extend to other motor tasks given that a simple key-pressing task was employed in the present study.

### Interpreting Higher Right Prefrontal Cortex Recruitment Following Repetitive Practice

The right prefrontal cortex is thought to be involved in skill acquisition as part of the frontoparietal motor learning network which also includes the right supplementary motor area and the posterior parietal cortex (Ziemann et al., 1995; Lin et al., 2012; Lage et al., 2015; Wright et al., 2016). Given this association with motor learning, increased right prefrontal cortex recruitment exhibited by the RP group during retention performance suggests that learners from the RP format had to further engage in motor learning processes compared to their IP counterparts. In other words, faster performance under RP came at a long–term cost (see also Immink and Wright, 1998). Repetition of the same task variation associated with low contextual interference afforded the learner the opportunity to bypass motor learning processes that would otherwise slow movement production. However, these learners then had to subsequently engage in the previously bypassed motor learning processes during the retention test performance. In contrast, IP engendered the development of motor learning processes resulting in retention test benefits in terms of both enhanced performance and reduced reliance on costly motor learning processes. Following RP, the requirement to engage more deeply in motor learning processes during retention incurred increased metabolic demands in the right prefrontal cortex such that neurovascular decoupling resulted in decreased levels of deoxygenated hemoglobin in this region (Tam and Zouridakis, 2014, 2015). That RP demonstrated substantial improvements in response speed between the first and second retention test block, while performance following IP was more stable, further illustrates the point that heightened right prefrontal cortex recruitment is indicative of increased motor learning.

### A Cognitive Control Account of the Contextual Interference Effect

Distinct left and right prefrontal cortex hemodynamic profiles between IP and RP groups at retention allows for further consideration of the effect of high and low contextual interference practice on prefrontal region recruitment. While the present study was not designed to specify what processes are associated with prefrontal cortex recruitment, consideration of these potential processes seems appropriate to contextualize the present fNIRS findings within the contextual interference effect. Recall, that the observed similarities in oxygenated and deoxygenated hemoglobin changes across practice were interpreted as representing comparable levels of prefrontal cortex recruitment between IP and RP. This would seem to contradict the main theme underlying the current theoretical explanations for the contextual interference effect—that more extensive motor preparatory and learning processes are engendered by IP. Specifically, IP would be expected to require greater prefrontal cortex recruitment due to more elaborate memory elaboration processes (Shea et al., 1985; Shea and Zimny, 1988) or action plan forgetting-reconstruction processes (Lee and Magill, 1983, 1985). Even as these perspectives are often viewed as describing processes that are more thoroughly engaged under IP, they might also provide an indication as to why the learner in RP might rely on prefrontal cortex neural activity.

According to both the elaboration (Shea et al., 1985; Shea and Zimny, 1988) and forgetting-reconstruction views, RP requires the learner to maintain in the working memory information that is specific to the task variation being performed in the block of trials. This information might relate to a perceptual or symbolic representation (Verwey et al., 2015) of the task variation that is held in working memory for a series of RP trials. In order to ensure that the salient information is maintained in working memory, the learner would need to rely on increased cognitive control.

Cognitive control refers to a set of information regulation processes, including attention narrowing and inhibition of interfering information, that are relied upon during goal-oriented behavior (Buschman and Miller, 2014; Amer et al., 2016), including sequence learning (Chan et al., 2017; Immink et al., 2017). Cognitive control is thought to be served by the frontoparietal network (Dosenbach et al., 2008) and the prefrontal cortex, in particular, has been associated with maintaining increased cognitive control (Koechlin et al., 2003; Badre et al., 2009; Buschman and Miller, 2014), particularly the dorsolateral prefrontal cortex (DLPFC: Goto et al., 2011). Thus, one potential interpretation of the present prefrontal cortex recruitment observed during RP is that it is reflecting reliance on increased cognitive control in order to maximize the benefits of low contextual interference in terms of speeded responding. The processes underlying prefrontal recruitment in RP would be markedly different to the prefrontal cortex-based processes employed under IP as in the latter case, prefrontal cortex recruitment is associated with motor learning processes (Jueptner et al., 1997). For example, prefrontal cortex activity during IP might allow for the conversion of a temporary perceptual or symbolic representation to a more enduring motor representation that serves long-term performance (Wright et al., 2004; Verwey et al., 2015; Immink et al., 2020). Evidence that IP and RP are engaged in different processes associated with the prefrontal cortex, despite the two practice formats exhibiting similar recruitment levels, is reflected in performance and prefrontal cortex recruitment observed in the retention test. Namely, RP resulted in increased recruitment in the right prefrontal cortex at test since the learner needed to implement the motor learning processes that were previously bypassed in practice conditions with low contextual interference.

### Limitations

The present findings need to be qualified with respect to limitations associated with preprocessing of NIRS data. Given that the motor task was performed in a stationary seated position, we did not expect any significant physiological influences associated with heartbeat, respiration, or blood pressure fNIRS signal. While there currently is no consensus on fNIRS data preprocessing for neuroimaging (Pfeifer et al., 2018; Klein and Kranczioch, 2019), a number of approaches have been proposed in the literature to reduce fNIRS signal contamination of cortical hemodynamic activity (Scholkmann et al., 2014; Khan et al., 2020) including bandpass filtering (Naseer and Hong, 2015; Kamran et al., 2016). The absence of bandpass filtering of the present fNIRS data does not allow us to rule out physiological noise as potentially contributing to our reported findings. However, the lower right PFC deoxygenated hemoglobin observed at test following RP, would appear to be a reliable effect since signal artifacts are thought to have a greater effect on the oxygenated hemoglobin signal (Zhang et al., 2016; Pfeifer et al., 2018).

## Conclusion

The present findings lend new insights into the processes underlying the contextual interference effect for motor learning. As proposed by Battig (1979), the contextual interference effect likely arises from a complex set of processes. The present focus on prefrontal activity, both during and following from IP and RP, has allowed us to highlight the involvement of processes that are potentially associated with but distinct from those described in the prevailing explanations (Shea and Morgan, 1979; Lee and Magill, 1983, 1985; Shea et al., 1985; Shea and Zimny, 1988) for the contextual interference effect. While prefrontal cortex engagement during IP has been previously described as contributing to the motor learning benefits (Kantak et al., 2010; Lin et al., 2011) from high contextual interference, the present findings suggest that prefrontal activity is also important for RP but for different purposes. Prefrontal cortex recruitment under RP might be associated with increased cognitive control (Jueptner et al., 1997; Dosenbach et al., 2008; Buschman and Miller, 2014) needed to maintain task variation information in working memory. While increased cognitive control might serve to enhance immediate performance under low contextual interference, it is not effective in supporting long-term performance as evidenced by poorer retention test performance following RP in contrast to IP.

## Data Availability Statement

The raw data supporting the conclusions of this article will be made available by the authors, without undue reservation.

## Ethics Statement

The studies involving human participants were reviewed and approved by Human Research Ethics Committee Charles Sturt University. The patients/participants provided their written informed consent to participate in this study.

## Author Contributions

MI, MP, and FM contributed to the study design, data analysis, and drafting of the manuscript. DW contributed to the data analysis and drafting of the manuscript. All authors contributed to the article and approved the submitted version.

## Conflict of Interest

The authors declare that the research was conducted in the absence of any commercial or financial relationships that could be construed as a potential conflict of interest.
